# Development of agroecology in Austria and Germany

**DOI:** 10.12688/openreseurope.15431.1

**Published:** 2023-02-02

**Authors:** Anna Brumer, Alexander Wezel, Jens Dauber, Tor Arvid Breland, Baptiste Grard

**Affiliations:** 1 Agroecology and Environment Research Unit, ISARA, Lyon, 69364, France; 2 Department of Plant Sciences, Norwegian University of Life Sciences, Faculty of Biosciences, Ås, NO-1432, Norway; 3 Thünen Institute of Biodiversity, Braunschweig, 38116, Germany; 4 Biodiversity of Agricultural Landscapes, Institute of Geoecology, Technische Universität Braunschweig, Braunschweig, 38106, Germany

**Keywords:** Agroecological practice, living lab, research in agroecology, agroecology movement, organic agriculture and food system.

## Abstract

**Background:** Transforming food systems is necessary to address the global issues of severe biodiversity loss, hunger, and malnutrition as well as the consequences of the rapidly advancing climate change. Agroecology as a systemic approach has been recognised as a promising path of change exemplified in various case studies strengthening this transformation. The aim of this study is to get insight specifically for Austria and Germany in providing an overview of the advancement in agroecology in both countries and identify agroecology-related initiatives.

**Methods:** 21 interviews with experts were conducted to determine the recognition, understanding, and development of agroecology in Austria and Germany in terms of movement, practice, policies, education, and research. In addition, information about agroecology-related initiatives was collected from interviews with 24 representatives of initiatives and literature analysis. Data was analysed according to five activity categories under which agroecology manifest: movement, practice, living lab, science and research infrastructure, and training and education.

**Results:** Results show that the term agroecology is not commonly used in Austria and Germany, where the concept is mainly associated to a scientific discipline. Practices considered agroecological are implemented primarily through organic agriculture, which is very developed in Austria and to a lesser extent in Germany. Many networks, food policy councils, associations, and scientific projects related to agroecology exist, each with specific purposes and ambitions to change farming and food systems. While most selected initiatives do not explicitly refer to agroecology, all follow certain agroecological principles and aim at contributing to accelerate the agroecological transition.

**Conclusions:** Clarifying the concept of agroecology, overcoming economic and political barriers as well as fostering participation of a multitude of stakeholders in the transition is essential for the future development of agroecology in Austria and Germany.

## Plain language summary

Agriculture in Europe is now facing increasing global challenges such as severe biodiversity loss, hunger, and malnutrition. This forces our societies to find ways to transform food systems to address those global issues. Agroecology as a systemic approach has been recognised as a promising path of change exemplified in various case studies strengthening this transformation. The aim of this study is to get insight specifically for Austria and Germany in providing an overview of the advancement in agroecology in both countries and identify agroecology-related initiatives. 21 interviews with experts were conducted to determine the recognition, understanding, and development of agroecology in Austria and Germany in terms of movement, practice, policies, and research. In addition, information about agroecology-related initiatives was collected from interviews with 24 representatives of initiatives and literature analysis. Data was analysed according to five activity categories under which agroecology manifest: movement, practice, living lab, science and research infrastructure, and training and education.

Our study highlights that the term agroecology is not commonly used in Austria and Germany, where the concept is mainly associated to a scientific discipline. Practices considered agroecological are implemented primarily through organic agriculture, which is very developed in Austria and to a lesser extent in Germany. Many networks, food policy councils, associations, and scientific projects related to agroecology exist, each with specific purposes and ambitions to change farming and food systems. While most selected initiatives do not explicitly refer to agroecology, all follow certain agroecological principles and aim at contributing to accelerate the agroecological transition.

Clarifying the concept of agroecology, overcoming economic and political barriers as well as fostering participation of a multitude of stakeholders in the transition is essential for the future development of agroecology in Austria and Germany.

## Introduction

Agroecology, understood as the ecology of farming and food systems ( Francis *et al.,* 2003), has since the 2000s increasingly been proposed as a useful concept to guide a much needed transformation of farming and food systems facing global issues such as severe biodiversity loss, hunger and malnutrition, poor agricultural resilience to the consequences of climate change and insufficient livelihood security for farmers ( Altieri *et al.,* 2015; Francis *et al.,* 2003; Gliessman, 2007; IAASTD, 2009; Wanger *et al.,* 2020; Wezel *et al.,* 2009). Agroecology addresses environmental, social and economic dimensions ( Altieri, 1989; Gliessman, 2018; Olson and Francis, 1995; Wezel *et al.,* 2009) by a holistic or systems thinking approach needed to understand the complexity and the interconnectedness of food system elements and processes ( Bezner Kerr *et al.,* 2019; Gliessman, 2016). Through its transdisciplinary, participatory and action-oriented approach ( Méndez *et al.,* 2016), agroecology aims to consolidate the links between the diversity of stakeholders (farmers, producers, researchers, and consumers) as well as those between different disciplines (ecology, agronomy, social sciences, economy, etc.).

Various challenges to accelerate an agroecological transition have already been identified ( Cacho *et al.,* 2018; Gliessman, 2019; IPES-Food, 2016; Wezel and Bellon, 2018). These include limited funding for agroecological research, lack of policies at the European Union (EU) level as well as weak connections between science, policymakers, and farmers. A further challenge linked to the latter, is the implementation of the agroecological principles ( HLPE, 2019; Nicholls and Altieri, 2018; Wezel *et al.,* 2020), which alongside systems thinking need to inform the selection and integration of concrete practices within the whole, e.g., a farming system. The need to generate, combine and exchange knowledge to reach cognitive justice, i.e., increase recognition of practice and give access to this knowledge ( Coolsaet, 2016) also plays a key role in the development of farming and food systems according to agroecological knowledge, principles and approaches ( HLPE, 2019; Wezel *et al.,* 2020). To overcome the aforementioned challenges and accelerate the transition, a long-term vison as well as a joint financial effort by the states are needed. A step towards this in Europe is the planned European partnership on agroecology living labs and research infrastructures ( https://research-and-innovation.ec.europa.eu/research-area/agriculture-forestry-and-rural-areas/ecological-approaches-and-organic-farming/partnership-agroecology_en).

Transforming food systems requires a series of steps, which may result in transition levels in increasing agreement with agroecological knowledge and principles ( Gliessman, 2016). Under the umbrella of agroecological systems thinking, the 13 principles of agroecology defined in the High Level Panel of Experts (HLPE) (2019) report, provide a basis which has to be adapted to the actual context and scale ( Wezel *et al.,* 2020). These principles range from those pertaining to agroecosystem components and partial perspectives such as soil health and animal health to broader system concepts such as synergy and connectivity of components and processes within and across ecological, economic, and social dimensions at various scales of farming and food systems. However, as an itemised list, they do not alone ensure the holistic, participatory, action-oriented systems approach, which forms the conceptual, ethical and methodological core of agroecology ( Gliessman, 2016; Méndez *et al.,* 2016).

At a national level in the EU countries, agroecology is not widespread but actions are currently undertaken regionally and locally, and there is a starting dynamic at EU level. France is an exception, where agroecology has found its way into legal texts and in public action already since 2014 ( Wezel and David, 2020). While European countries differ in their approach to agroecology, it was reported that most conceive agroecology firstly as a science, then as a practice and to a smaller extent as a movement ( Gallardo-López *et al.,* 2018). In this regard, several sources or databases on agroecology and agroecology-related initiatives, with different objectives, already exist. The Agroecology Knowledge Hub ( https://www.fao.org/agroecology/home/en/), a web platform created by the FAO, shares relevant knowledge, documents and policies (AgroecologyLex) on agroecology around the world. In recent years, the importance of mapping and setting up databases on agroecology has been recognised, as “mapping has an important role to play in strengthening processes of transformation” ( Milgroom *et al.,* 2019). Different agroecology-related initiatives, constituting examples of successful practices in farming and food systems in nine European countries, are presented by Moraine *et al.* (2016), who also analysed their performance (production, economic, farm autonomy, work management, inputs self-sufficiency, domestic biodiversity, and landscape diversity). A special journal issue around the manifestation of agroecology in Europe ( Wezel and Bellon, 2018) gave a first insight into what is happening in different countries. This includes a first analysis for Mediterranean countries ( Migliorini *et al.,* 2018), eastern Europe ( Moudrý *et al.,* 2018), and Belgium ( Stassart *et al.,* 2018) It was followed by a report of Agroecology Europe (2020), which mapped initiatives in 11 European countries. Subsequent publications provided analyses of the current state of agroecology-informed initiatives and a mapping of such initiatives in Hungary ( Balogh *et al.,* 2020) and in the West Balkans ( Šeremešić *et al.,* 2021). These publications show that a multitude of initiatives and projects exist with different approaches on various themes such as education, commercialisation, production and food sovereignty. However, most of them do not cover all relevant elements and dimensions of the agroecosystem, let alone take a holistic, systems- and action-oriented approach in the pursuit of overall food system sustainability ( Wezel *et al.,* 2009).

This study was part of the Horizon2020 Agroecology for Europe ( www.ae4eu.eu) project aiming to map European initiatives and development in agroecology in different countries in Europe. The aim of the study was to map initiatives linked to agroecology and analyse their current state in two European countries: Austria and Germany. The term mapping is understood here as a collection of information on existing initiatives, e.g., examples of innovative projects or associations pursuing the improvement of agriculture. Analysing state here means assessing their main area of action (movement, practice, living lab, science and research infrastructure, and training and education) and stage of development. Having an updated analysis of the current state of agroecology-related initiatives in Austria and Germany in terms of recognition, understanding, and implementation will provide an overview that can serve European policies for developing sustainable food system. Favourable policies are key drivers to scale out practical manifestations of agroecology ( Cacho *et al.,* 2018). The objective of this study was to answer the following questions:

1 What is the current recognition and understanding of agroecology, in movement, practice, living lab, science and research infrastructure, and training and education in Austria and Germany?
2 How are existing initiatives contributing to the implementation and development of agroecology in Austria and Germany?

To answer these questions, experts and representatives of relevant initiatives were interviewed, and online research and a literature study were conducted.

## Methods

### Ethical statement

For each interview – of key and initiative informant - the interviewee received in advance an informed consent form as well as information document with all relevant information. All interviewees were asked to sign consent forms prior to the interview. If necessary, relevant information were explained orally at the beginning of the interview. No relevant ethical issues were identified by AE4EU project regarding human intervention in the proposal. For the collection of personal data, detailed information on the procedures for data collection, storage, protection, retention, and destruction, and confirmation that they comply with national and EU legislation are described in the deliverable D8.2 submitted in March 31,2021 and accepted by the EU Commission. The project Ethics committee, consisting of the project coordinator, the data protection and management officer, as well as representative of selected partners of the Ethics work package validated the questionnaires used in the surveys of this study. Written informed consent was also obtained from participants to the surveys to use their answers and quotations for research and publication.

### Manifestations of agroecology

**Figure 1.**
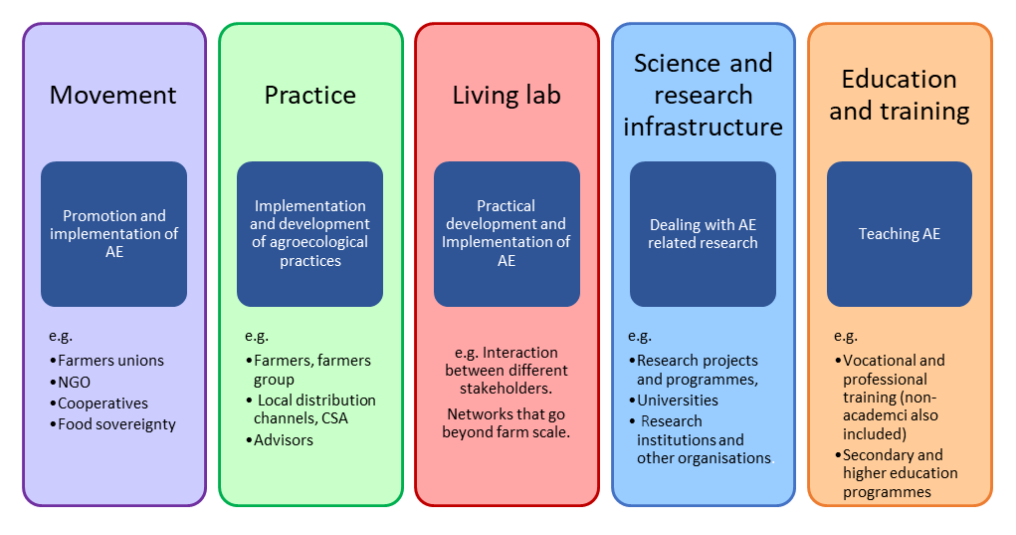
The five activity categories mapped in Austria and Germany for manifestations of agroecology (AE).

We investigated the historical development and current occurrence and status of agroecology as manifested in the following activity categories: movement, practice, living lab, science and research infrastructure, and training and education ( Figure 1). Associations, civil society stakeholders, non-governmental organisations (NGOs), and farmers’ unions promoting the application of agroecology were considered as movements. Practices included farmers or any stakeholder that develop and implement agroecology. Research projects and programmes, universities and institutions doing agroecology-related research were considered for the science category. Training and education is an activity area that is often integrated into science, however, it was considered here as a separate category as it also includes trainings that are done outside of academic settings and research infrastructures, for example, by NGOs. Finally, living labs follow the definition of being open innovation networks involving a multitude of actors ( Dekker *et al.,* 2020; Leminen, 2015), beyond the farm scale, and implementing and developing agroecological principles. These types of initiatives are often very recent and may represent an important supporting pathway for an agroecological transition in Europe.

### Methodological steps of the interview-based research

**Figure 2.**
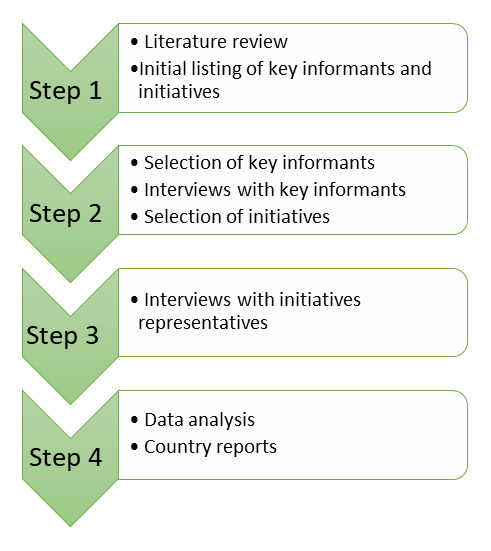
Main methodological steps for mapping agroecology in European countries.

The five methodological steps used to map manifestations of agroecology in Austria and Germany are summarised in Figure 2. The first step consisted of a literature review regarding the historical development and current status of agroecology in the two countries and of collecting current information on agroecology in both countries by searching on government and initiatives websites.

**Figure 3.**
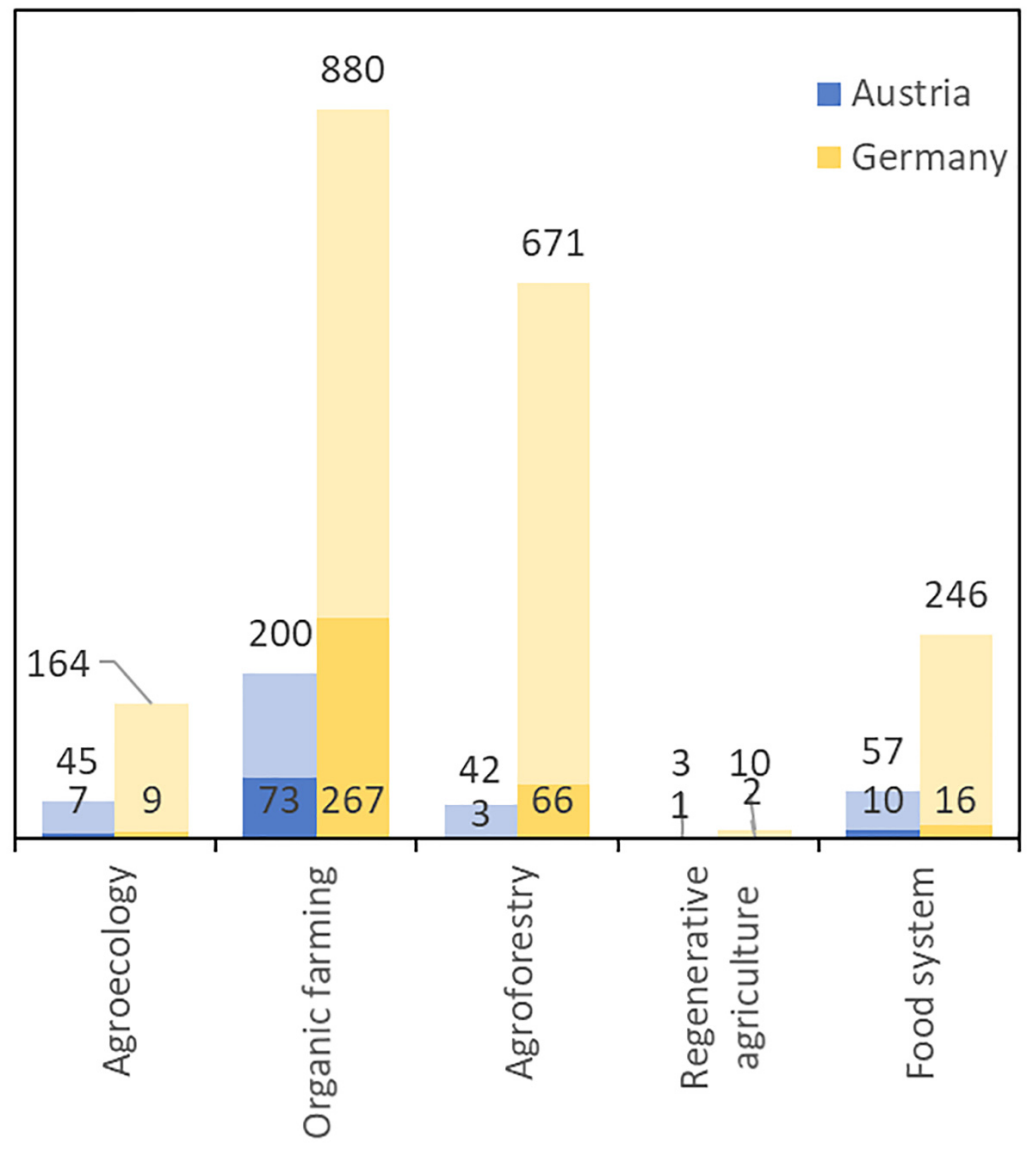
Publications from 1990 to 2021 with Austrian and German authors involved using five themes and related keywords as a topic: 1) agroecology, 2) organic farming, 3) agroforestry, 5) regenerative agriculture, and 6) food system. The columns represent the total number of articles per topic and the data colours indicate that the country was included in the topic. Darker yellow or blue concern the study regarding including Germany and Austria as a topic.

**Figure 4.**
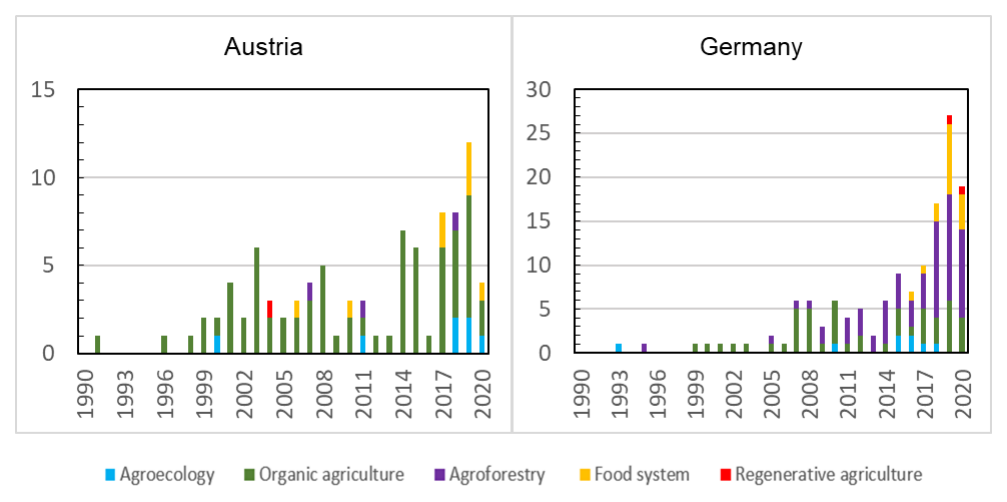
Publications from 1990 to 2021 with Austrian and German authors involved using five themes and related keywords as a topic: 1) agroecology, 2) organic farming, 3) agroforestry, 5) regenerative agriculture, and 6) food system. The columns represent the total number of articles per topic and the data colours indicate that the country was included in the topic. Darker yellow or blue concern the study regarding including Germany and Austria as a topic.

The literature review and an analysis of academic publications in Austria and Germany was undertaken on the ‘Web of Science’ platform, using the keywords i) agroecology and agroecological farming, ii) organic agriculture, organic farming, organic horticulture, organic livestock and biodynamic, iii) agroforestry, silvopasture, and silvoarable, iv) regenerative agriculture, regenerative farming and permaculture, and v) agroecology territories related keywords such as food justice, food systems, food sovereignty, and rural development. The country name was also included in the topic search to investigate the number of published articles where one of the contributing authors was a researcher in Austria or Germany, and second, the number of published articles where the article focuses on research carried out inside the respective country. Articles between 1990 and April 2021 were considered. All scientific articles in German and/or English found were included in the analysis. Each article was read and categorized per year of publication and according to key words category (see Figure 3 and Figure 4).

In the internet search for current information, we used the words “agrar(-)ökologie” and “biologischer Landbau” or “ökologischer Landbau”. We included organic agriculture into the search, as organic agriculture, at least ideally, reflects agroecological knowledge, worldview and methodological approaches to systems development and selection of practices ( IFOAM, 2019; Migliorini and Wezel, 2017). This data collection was complemented by data from a first screening of European agroecology livings labs and research infrastructure initiatives launched by the DG-Agri in 2020 ( https://ec.europa.eu/eusurvey/runner/FirstScreeningAELLRI2020). Based on this, a first selection of key informants and initiatives was established.

The second step consisted of interviewing key informants, from an initial listing of step 1 and expert knowledge, with a semi-structured questionnaire (see the description below). The selection of further key informants and initiatives was based on the interviews with the initial key informants. Some key informants were also involved in initiatives; in such cases, the interview continued collecting information about the initiative.

In the third step the obtained data was analysed and finally in the fourth step a country report was produced which including a description of the different initiatives, which is not part of the present paper.

### Selection and interviews of key informants and initiatives

Key informants were selected based on their knowledge on agroecology within one or more of the five activity categories ( Figure 1) and representing different institutions and organisations. These included individuals having participated in national gatherings or conferences about agroecology, in previous mapping projects, or being researchers at universities or institutes with a focus on agroecology. Representatives of NGOs and civil society organisations active in agroecology and food sovereignty, as well as members of chambers of agriculture (e.g., in the organic farming sector) were also selected, in addition to those identified in the DG-AGRI survey. Key informants were also asked to name other experts. The initiatives were then selected according to the following criteria: being named by more than one key informant and having objectives in line with at least one of the 13 principles of agroecology ( HLPE, 2019). They further had to be viable and have existed for at least three years (with possible exception for outstanding initiatives and recently created living labs).

As agroecology is not a term that is commonly used in Austria and Germany, most initiatives selected did not label themselves specifically as agroecological, but all were using one of the keywords used to find relevant publications for the literature review and web search. A further selection criterion was the localisation; an effort was made to find initiatives in different regions of both countries.

### Interviews

The interviews followed a semi-structured questionnaire in English developed for mapping manifestations of agroecology in Europe. The questionnaire was developed by AE4EU partners between January to March 2021, based partly on a previous study carried out by Agroecology Europe (2020). It was internally translated to German for this study, but two interviews were, nevertheless, conducted in English. The interview of key informants ( Grard *et al.,* 2023) started with a question about how often the key informants used the term agroecology and what their definition of it was. This was followed by a series of questions on their knowledge of initiatives within the five activity areas. The last part of the interview consisted of questions on awareness, policies, practices used, and barriers as well as opportunities for the development of agroecology. Key informants’ interviews lasted between 30 to 70 minutes. Each interview was recorded and key findings transcribed into a database ( Grard *et al.,* 2023). In the database, key informants were associated with a number (key-informant 1, 2, 3 etc.) to anonymise the data collected.

In interviews with the selected initiatives, a second semi-structured questionnaire was used ( Grard *et al.,* 2023). It included questions on starting year, involved stakeholders and future plans of the initiative as well as funding sources and regional–national representation. Those interviews lasted 30–45 minutes. Each interview was recorded and key findings transcribed into a database ( Brumer *et al.,* 2023) frame according to interview question.

### Data analysis

Data from the interviews ( Brumer *et al.,* 2023) were analysed to establish an overview of the current state of agroecology-related initiatives in the respective country within the five activity categories presented in Figure 1). This included the awareness within the civil society, the level of integration in political directives at national and regional level, the existing educational programmes and research projects, and the supporting factors, challenges, and barriers for the development of initiatives informed by agroecology in the country. Data were summarised and analysed using a standardised excel data base also used for mapping agroecology initiatives in other European countries. The transcripts of the interviews with key informants were also analysed with a statistical tool from R-4.1.0 ( https://www.r-project.org/) for extracting the frequency of relevant keywords using the “tm” tool.

## Results

Interviews were carried out with a total of 21 key informants and 24 initiatives: 13 key informants and 15 initiatives in Germany, and eight informants and nine initiatives in Austria, respectively. Interviews were held between March and June 2021. In Austria, half of the key informants were working at chambers of agriculture ( Table 1) in different regions. In Germany, the majority of key informants were working at universities or research organisations ( Table 1).

**Table 1.** Key informants interviewed in Austria and Germany.

Country	Number of interviewees	Type of structure	Dimension of agroecology
Austria	4	Chamber of agriculture	Practice
	1	Research organisation	Science, living lab, education and training
	1	NGO	Movement, practice, science
	1	University	Education and training, science
	1	Ministry of agriculture	Practice, science
Germany	4	University	Education and training, science
	1	NGO	Movement
	4	Research organisation	Science, education and training, living lab (only one key informant)
			Science
	2	Ministry of agriculture	Science
	2	Chamber of agriculture	Practice

We first present here the historical development of agroecology-related initiatives and terminology, the key informants’ view on agroecology and its definition, existing policies and practices related to agroecology, and we then present data about publications on agroecology-related topics since 1990. This is followed by a section focusing on existing initiatives within the five activity categories described above. Τhe last section describes the barriers and opportunities for the further development of agroecology-related initiatives.

### Historical development and current occurrence of agroecology-related initiatives and terminology in Austria and Germany

Historically, Austria is a pioneer country in organic farming, starting with the development of biodynamic agriculture by Rudolf Steiner in 1924. Τhe first biodynamic farms were created in 1925 in Carinthia, and the first organic association (which later became Demeter) was established in 1932 ( Steinwidder and Starz, 2016). Organic agriculture was promoted by the Bio-Aktionsprogramm 2015–2020 (action programme for organic farming), promoting key measures to further develop organic farming ( Rech, 2015), which has been prolonged until 2022. Another element of this programme is the high allowance payments to organic farms in less favoured areas and the “Biobonus” (i.e., higher subsidies for organic farming). The Austrian agri-environmental programme Österreichisches Programm für Umweltgerechte Landwirtschaft (ÖPUL), supports amongst others water conservation measures, biodiversity conservation, integrative pest management, and organic agriculture (over 40% of its budget goes to organic agriculture). It can therefore be considered as a programme promoting the implementation of agroecological knowledge, worldview and approaches in practice. A specificity of Austria is that mountainous areas make up 70% of its surface area and according to EU classification (Art. 32(2), Regulation 1305/2013) they are considered as disadvantaged regions. Austria has the largest area of organic farmland in the EU and third worldwide ( Steinwidder and Starz, 2016). Over 25% of the agricultural land in Austria is farmed organically (as of 2019), and 22 % of farms are certified organic ( BMLRT, 2020).

Germany has a similar development of organic agriculture as Austria, but with a smaller share of organic land and farms. In 2019, around 10% of the farmland in Germany was farmed organically ( BMEL, 2021) and 12.9 % of the farms were certified organic. For Germany, the position paper “Agrarökologie stärken — Für eine grundlegende Transformation der Agrar- und Ernährungssysteme” ( INKOTA, 2019), called the German federal government to take a series of measures supporting agroecology-related initiatives. These include specific financial support as well as the development of farmer-led research, principles of co-creation of knowledge used in research and the publication of a progress report every two years. The report of Haller *et al.* (2020) outlines development perspectives for organic farming and how organic and conventional agriculture could be optimised.

In Austria and Germany, organic agriculture often goes well beyond the European organic standard, as expressed in many established association guidelines such as Demeter, Bioland, Naturland, and BioAustria. All key informants in Austria agreed that in their principles and practices, they see a close relation between organic agriculture and agroecology, whereas in Germany agroecology was often seen as a broader subject and approach but in close agreement with the principles of organic agriculture.

### Definitions, perceptions, and development of agroecology

***Definitions.*** In Austria, only one out of eight key informants reportedly use the term agroecology very often, three often, and five rarely in their respective work. In Germany, five out of 13 reported using it very often while four often, and four rarely.

When asked about their definitions of agroecology, most key informants (five) in Austria defined it as a practice for sustainable production, meaning not negatively impacting the environment. Three defined it as a scientific discipline studying the interactions and relationships in an ecosystem of which two added that it is also a political movement. For Germany, key informants mainly defined the concept as a science (10), with four also mentioning it being considered as a social movement and three using the threefold definition by Wezel *et al.* (2009). Some key informants argued that it is a holistic and systemic approach (two in Austria, four in Germany).

Organic farming was also mentioned in the definitions, with two key informants in Austria specifying that organic farming is the implementation of agroecology and two informants in Germany stating that agroecology is based on the principles of organic farming or includes organic farming. Three informants in Germany also insisted on the notion that agroecology represents a transformative process towards a sustainable food system.

***Agroecology-related terms mentioned by the interviewees.*** Not counting the words agroecology and initiative, the most repeated words during the key informants’ interviews in Austria, were farms (“Betriebe”), agriculture (“Landwirtschaft”), farmer (“Landwirt”), organic agriculture (“Biolandbau”), organic (“biologisch”), and measures (“Maßnahmen”). In Germany, they were agriculture (“Landwirtschaft”), measures (“Maßnahmen”), farmer (“Landwirt”), biodiversity (“Biodiversität”), transformation (“Transformation)”, and organic farming (“Ökolandbau”).

The word ‘measures’, frequently repeated in both countries, was most often linked to agri-environmental measures but sometimes also to nature or climate protection measures. “Consumer”, “society” and “research” were also repeatedly mentioned by the key informants in Germany to play an important role in food systems. In Austria, “BioAustria” and “ÖPUL” were frequently repeated, showing the importance of the organisation and agri-environmental programme for the development of sustainable food systems.

***Policies related to agroecology.*** In Austria, key informants, referring to the ÖPUL stated that there are already policies helping the implementation of agroecology in practice. Other policies mentioned were the EU organic regulations (EG - Nr. 834/2007 and Nr.889/2008), the common agricultural policy (CAP - specifically the agri-environmental schemes in the 2 ^nd^ pillar) and the association guidelines from BioAustria, Demeter, or Bioland. BioAustria, which represents two thirds of all organic farmers in Austria, has guidelines going beyond the organic farming regulations. For example, all produce of a farm needs to be organic to have the BioAustria label. Other major differences to the EU organic regulations and label are on animal welfare requirements. There are also requirements that are not mentioned in the EU regulations on packaging, horticultural production, communication and education.

The response to the existing policies regarding agroecology in Germany varied. Half of the key informants answered negatively to the question if there are any policies helping the implementation of practices according to agroecological principles, either by saying not at all or not really. Most agreed that the focus of existing policies was not on agroecology. On top of the CAP and EU organic regulations, different strategies and policies such as the Biodiversity Strategy (“Biodiversitätsstrategie”), livestock strategy (“Nutztierstrategie”), arable farming strategy (“Ackerbaustrategie”), the fertiliser regulation (“Düngeverordnung”) as well as the recent insect protection law (“Insektenschutzgesetz”), and the nature conservation agreement (“Naturschutzvertrag”) were mentioned in Germany. These strategies and policies have some goals and practical measures, e.g., limiting the amount of fertilizer and protecting specific species, that are agroecologically favourable. In an agroecological perspective, though, they require a holistic systems approach to be integrate with other measures taken in the pursuit of overall ecological, economic and social sustainability.

***Implementation of practices and farming systems.*** To get an overview of the implementation in both countries of practices that may be compatible with agroecological knowledge, worldview and approach, the key informants were asked to name examples of the most commonly used practices. While some are clearly defined practices, a few, such as organic farming, refer to a production system which includes a conglomerate of practices. Other practices mentioned were linked to agri-environmental measures, such as flower strips, which can be established for different purposes, e.g., supporting natural enemies in order to reduce the application of insecticides. Crop rotation and organic farming for Austria and flower strips and organic farming for Germany were the most mentioned practices. All interviewees could not give any estimate to the frequency of use of these practices. One informant specified that while flowering strips are very common, they probably only represent 1% of agricultural surfaces in Germany when comparing it to the amount of organic certified agricultural surfaces, which is 10% of agricultural surfaces; “organic farming is by far the most common practice” (Key informant 12 – Germany; Grard *et al.,* 2023).

### Science and publications

When looking at published articles (in English) with keywords related to agroecology, the highest number employ the concept of organic agriculture. 1,080 articles using organic agriculture as a topic were published during the last 30 years with at least one author from an Austrian or German research institution or organisation. 209 articles with agroecology as a topic were published by authors working in either country, which is less than the 303 articles related to food systems. For Germany, a very high number of articles related to agroforestry were published (671).

The number of articles also including the country as a topic ( Figure 3 and Figure 4) is noticeably lower in all five selected terms for both countries, showing that the experiments or focus are either based outside of Austria or Germany or that possible articles are not based on empirical data. Articles on organic farming represent 29% of the papers published on agriculture in Austria and 21% in Germany during the period from 1990 to 2021. During the last five years, they represented 27% for Austria and 20% for Germany.

The first scientific article (in English) on agroecology in Germany was published in 1993, for Austria it was in 2000 ( Figure 4). The publication of articles on agroforestry and food system in Germany has increased in the last ten years. An increasing trend can also be seen for the articles on agroecology in Austria since 2018. At least one article on organic agriculture in Austria and Germany was published every year starting from 1996 and 1999, respectively. Only one article on the topic of regenerative agriculture in Austria was published so far (in 2004), whereas two were published for Germany (in 2018 and 2020).

### Initiatives in Austria and Germany

The aim and general characteristics of the 24 selected initiatives are summarised in Table 2.

**Table 2.** Agroecology-related initiatives in Austria and Germany.

**In Austria: **					
Initiative name	Scale	Stakeholders	Founded in	Aim	Related activity category
*Feld - association of the use of unused* *("Verein von Nutzung von Ungenutztem")*	Local	Civil society, farmers	2014	Reducing food waste by transforming unsold food	Movement
*Arche Noah*	International	Civil society	1989	Preservation and development of the diversity of cultivated plants	Movement, education and training
*Vienna Food Policy Council* *("Ernährungsrat Wien")*	Local/National	Civil society	2018	Relocating – food system and decision making processes in Vienna	Movement, education and training
*Results oriented nature conservation* *("Ergebnisorientierter* *Naturschutzplan")*	National	Farmers, advisors	2012	Result based nature conservation planning	Practice, education and training
*Grand Farm*	Local	Farmers, researchers	Organic since 2006	Innovations along three themes: soil health, agroforestry, market gardening	Living lab, practice, science
*Long Term Ecological Research* *(LTER)*	National	Researchers, farmers	2002	Long term ecological research plots	Living lab, science
*Biodiversity monitoring with farmers* *("Biodiversitätsmonitoring mit* *LandwirtInnen")*	National	Farmers, researchers	2007	Farmers monitoring biodiversity in agricultural landscapes, changing practices to promote biodiversity	Education and training, practice
*Bioschool Schlägl ("Bioschule* *Schlägl")*	Local	Students	2002	Organic agricultural school (14–17 years old students)	Education and training
*Permaculture Academy (PIA – "Permakultur* *Akademie im Alpenraum")*	National	Civil society	2004	Teaching permaculture (all ages)	Education and training
**In Germany:**					
Initiative name	Scale	Stakeholders	Founded in	Aim	Related activity category
*Aktion Agrar*	National	Civil society	2014	Actions for agricultural turnaround	Movement, education and training
*German professional association* *agroforestry (DeFAF)*	National	Civil society, farmers, researchers	2019	Promote agroforestry in Germany	Movement
*Food PolicyCouncil Frankfurt* *("Ernährungsrat Frankfurt")*	Local/National	Civil society	2017	Promote regional, fair and ecological food supply, involve civil society	Movement, practice, education and training
*Model eco-regions ("Ökomodellregionen* *Bayern")*	Regional	Civil society, farmers, advisors	2014	Increase organic production, create regional value chain	Practice
*Grassland biotope network* *("Biotopverbund Grasland")*	Regional	Researchers, farmers, advisors, civil society	2017	Create and maintain biotopes in grassland	Practice
*Demonstration network for pea and bean* *("DemoNet Erbse Bohne")*	National	Farmers, researchers	2016	Support cultivation and processing of beans and peas in Germany, linking demand and supply	Practice, living lab, science
*Network for animal wellbeing ("Netzwerk* *Fokus Tierwohl")*	National	Farmers researcher	2019	Animal welfare, environmentally friendly and sustainable livestock farming	Practice, living lab, science
*Network for stock protection ("Vorratschutz* *Netzwerk, Vsnet")*	National	Researchers, farmers	2019	Sustainable post-harvest protection	Practice, living lab
*Biodiversity model farmsin North-Rhine* *Westphalia ("Leitbetriebe Biodiversität")*	Regional	Farmers, advisors	2015	Implementation and adaptation of agri- environmental measures	Practice, living lab
*patchCROP*	Regional	Researchers, farmers	2019	Increase agricultural diversification by temporal and spatial approaches at the landscape level	Living lab, science
*F.R.A.N.Z.*	National	Researchers, farmers	2016	Implementing effective biodiversity promoting measures	Science, practice, living lab
*Biodiversity Exploratories*	Regional/ National	Researchers, farmers	2006	Fundamental ecological research in selected large-scale areas	Science, practice
*Agriculture management and biodiversity* *("Agrarmanagement und Biodiversität")*	National	Students	2018	Master course for future biodiversity advisors	Education and training
*Bridging generations in* *agroecology*	National	Farmers, students	2020	Development of suitable seminars and courses on agroecology for farmers	Education and training
*Acker e.V. – Vegetables academy* *("GemüseAckerdemie")*	International	Students (pre-school, school)	2014	Strengthening awareness of the importance of nature and the appreciation of food	Education and training, practice

***Movements.*** The concept of agroecology has been used by different movements in both countries, even if the term itself is not always explicitly used. Movements are often linked to food sovereignty (e.g., ÖBV-via Campesina Austria, Nyéléni Austria) and Community Supported Agriculture (e.g., CSA, Solidarische Landwirtschaft in both countries). Over 40 initiatives of CSA have been listed in Austria ( https://www.ochsenherz.at/solidarische-landwirtschaft-in-oesterreich-2/) and over 362 in Germany ( https://www.solidarische-landwirtschaft.org/solawis-finden/karte#/). 

Another type of citizen-led movement is the emergence of food policy councils aiming to involve citizens in decision processes in food systems ( Sieveking, 2019), and thereby creating a new appreciation for food and its producers, promoting local, sustainable and fair food supply. The Vienna food policy council ( https://ernaehrungsrat-wien.at) follows sociocratic principles in decision making processes, meaning that every member can express their ideas and opinions on specific proposals, and decisions are taken in groups. Around 40 people are active in the different projects including the development of a food strategy (named “Ernährungsstrategie”) with the city of Vienna and an urban field (called “WeltTellerFeld”) representing the yearly food consumption per person and the necessary surfaces of arable land and pasture needed to provide all food products. The food policy council in Frankfurt ( https://ernaehrungsrat-frankfurt.de) has a similar structure and has several working groups on education and awareness raising, production and marketing, zero waste and permaculture. The number of people actively involved is fluctuant. Currently about 150 people are involved. Both food policy councils follow agroecology principles ( HLPE, 2019) such as recycling (food waste), co-creation of knowledge, social values and diets, connectivity and participation. Their work is based on volunteers. A difficulty mentioned by both initiatives is the lack of recognition and financial support by governments.

***Practice and living labs.*** In Austria and Germany different regions have been labelled as ‘organic model regions’, their common objective is to increase the production of organic food and create short supply chains with the involvement of municipalities and different stakeholders of the food system. The “Ökoregion Kaindorf”, the BioRegion “Mühlviertel” in Austria, and the different “Öko-Modelregionen” in Bavaria and Hessen, as well as the “Öko-Musterregionen” in Baden Württemberg, were considered as examples of implementation of agroecology.

Four living labs, identified in the DG-AGRI survey and interviewed for the purpose of this study are: the Grand Farm ( https://grandfarm.at), the long-term field experiments of the AGES (Austrian Agency for Health and Food Security), patchCrop ( https://comm.zalf.de/sites/patchcrop/SitePages/Homepage.aspx) and the Biodiversity Model Farms in Nordhrein-Westfalen ( https://www.landwirtschaftskammer.de/landwirtschaft/naturschutz/leitbiodiversitaet/index.htm). All involve different stakeholders (farmers, advisors, researchers) and aim at transforming or adapting practices. They differ regarding the process of co-creation of knowledge. Indeed, in the patchCrop project the farmers and researchers co-designed the experiment, whereas for the model farms the agri-environmental measures are proposed by the advisors and then implemented by farmers.

Other initiatives included in the practice and living labs activity category as a main area of action include initiatives such as the “Biotopverbund Grasland” ( https://www.gruenlandzentrum.org/projekte/biotopverbund-grasland/), “DemoNet Erbse Bohne” ( https://llh.hessen.de/pflanze/eiweissinitiative/demonstrationsnetzwerke/demonstrationsnetzwerk-erbse-bohne/), “Netzwerk Fokus Tierwohl” ( https://fokus-tierwohl.de/de/) and “Voratschutz Netzwerk” ( https://www.netzwerk-vorratsschutz.de/vsnet/de/home). They could also be considered as living labs, as these networks link many different stakeholders to a common objective of increasing biotope connections, animal welfare and reducing the synthetic inputs for the post-harvest protection. The motivation behind the creation of these networks is not just the demonstration of different practices but the adaptation and idea exchange on the different practices, which is subsequently assessed by scientists before being disseminated nationally or regionally through guidelines or policies.

***Science, education, and training.*** The science of agroecology integrates a multitude of subjects and is often fragmented in different research areas in Austria and in Germany ( Table 3 and Table 4). The most often stated universities were the University of Life Science (BOKU) in Vienna, University of Göttingen and University of Hohenheim in Germany. The BOKU and the University of Hohenheim offer together with other European partners a joint Master of Science (MSc) in Organic agriculture and food systems (EUR-Organic), and at Hohenheim also as single degree with the same name and at BOKU as Agroecology-Organic agriculture. The other universities listed in Table 3, have all groups or departments working on agroecology-related subjects and offer various related courses, but they are rarely named agroecology and they focus on specific topics such as soil health, animal health and wellbeing. Other research institutions and research infrastructures were also mentioned by key informants, some are federal institutions. Only the Institut für Agrarökologie und Biodiversität (IFAB) in Mannheim focuses specifically on agroecology while others like Bioforschung Austria and Research Institute of Organic Agriculture (FiBL), concentrate their research on organic farming.

**Table 3.** List of universities with a department or a research group or unit related to agroecology in Austria and Germany.

University	Research topic
**Austria**	
BOKU - Vienna	Sustainable agricultural systems (agroecology and organic agriculture)
Innsbruck	Agricultural and regional sociology

**Germany**	
Göttingen	Agroecology; Functional Agrobiodiversity
Hohenheim	Ecology of Tropical Agricultural Systems; Landscape ecology
Kassel - Witzenhausen	Organic agricultural sciences
Freiburg	Nature conservation and landscape ecology
München - Weihenstephan (TUM)	Life science systems
Humboldt - Berlin	Agricultural and food policy
Giessen	Animal ecology
	Landscape ecology
Kiel	Landscape ecology
Bonn	Economics of Sustainable Land Use and Bioeconomy; Agroecology and organic agriculture
Lüneburg	Ecosystem functioning and services lab
Greifswald	Landscape Ecology and Ecosystem Dynamics
Münster	Applied landscape ecology and ecological planning
Koblenz - Landau	Ecosystem analysis
Cottbus-Senftenberg (BTU)	Organic pest management;
	Social science environmental issues

**Table 4.** List of research institutions and infrastructures in Austria and Germany carrying out research related to agroecology.

Country	Research institutions and research infrastructure
**Austria**	Bioforschung Austria
	HBLFA Raumberg-Grumpenstein - Higher federal teaching and research institute for agriculture
	AGES - Austrian Agency for Health and Food Safety

**Germany**	Thünen Institute
	UFZ Helmholtz - center for environmental research
	ZALF - Leibniz Centre for Agricultural Landscape Research
	JKI - Federal Research Centre for Cultivated Plants
	Biodiversity exploratories
	IFAB - Institute for Agroecology and Biodiversity
**Both countries**	FiBL - research institute of organic agriculture

### Future development of agroecology

Key informants were asked to identify the barriers and opportunities to further develop agroecology-informed initiatives in Austria and Germany.

***Barriers.*** For both countries, economic barriers were the first and most mentioned barriers for future development of agroecology. These included the inadequate funding schemes, which do not really promote the implementation of agroecology, the insufficient remuneration of farmers, and high labour costs. Stakeholders in Germany mentioned that there is a lack of cost/benefit analyses demonstrating that agroecology is not only key to handle many environmental problems but also an approach that may provide economic benefits in the long term. A further barrier mentioned was the influence of the agribusiness lobby. Economic barriers are closely linked to political barriers, with a lack of incentive to develop and implement biodiversity-promoting measures, and to consider farms, farmers and the environment, including the consumers, as an interconnected whole. The administrative burden is perceived as a discouraging factor for the implementation of agroecology-informed production systems.

The third type of barriers is linked to the awareness and education of civil society, including farmers. Food prices were recognised as being too low in both countries as they do not account for the environmental externalisation of costs. In order to change this, some key informants argued that consumers need to become aware and ready to pay true costs, whereas others claimed that more financial means from the states or the EU could change this. While the conflict between nature conservation organisations and farmers was mentioned by most key informants in Germany, only one referred to this as being a barrier for the development of agroecology-related initiatives. For Austria, two key informants believed that the biggest hurdle is the land use, as it becomes more profitable to use the land for energy production than food production and land pressure is rising because of soil sealing. Another barrier mentioned, was the gap between scientific knowledge and implementation. Two informants pointed out that scientific knowledge is missing to allow a proper and practical implementation of agroecology. The definition of agroecology as being perceived by key informants remains unclear and very broad. Key informants for Germany see it as the first barrier to be overcome. Finally, one key informant stated that the main barrier is the difficulty to completely change the system and get out of lock-ins to truly accelerate the transition to agroecologically sound, more sustainable farming and food systems.

***Opportunities.*** The majority of key informants agreed that the time is ripe for practical manifestations of agroecology and that there is a real momentum in both countries. The trend of consumers asking for local and sustainable products has been accentuated during the COVID-19 pandemic. More and more people become aware of the climate change threat and the loss of biodiversity. This leads to a certain change of consumption habits and a readiness to more strongly support farmers, e.g., those practicing organic agriculture. Bottom-up movements are increasing and the notion of living labs was seen as very promising by the few key informants who already familiar with the concept. Different ideas for the development of agroecology-informed practice(s) were raised, starting with the improvement of the image of agriculture, reconnecting consumers to producers and the need to demonstrate the viability of farming and food systems based on agroecology. Another proposition was the recognition of the ‘production of biodiversity’ as an agricultural branch, similar to the energy production branch developed in recent years. A recommendation was the necessity to include all farmers, organic and conventional, and promote cooperation with all stakeholders involved in a territory, to remedy the too often opposing formed by nature conservationists and farmers. The last recommendation concerned the further development of organic agriculture and the risk of developing agroecology in parallel when it is in fact compatible to the notion of organic agriculture (AT key informant). For Germany, key informants addressed the fear that agroecology as it is not clearly defined and understood could weaken the high standards of organic farming and further play into the confusion of consumers. The most considerable opportunity for agroecology is to link food system stakeholders and to foster cooperative and bottom-up movements.

## Discussion

### Recognition and understanding of agroecology in Austria and Germany

The concept of agroecology is only recognised by few stakeholders in Germany and Austria, and it is understood differently among the interviewees of the present research. Finding key informants and initiatives proved to be difficult, as the word itself (“Agrarökologie”) is not commonly used. Agreeing on a definition of agroecology remains a key task for its recognition in Germany where it is still mainly seen as a science as discussed by Wezel and Soldat (2009). The definitions given by key informants reflected their work, researchers and professors always defined agroecology as a science while advisors in the chamber of agriculture focused on the practice.

Another explanation for the lack of recognition of agroecology is the historical development of organic agriculture in both countries, which is the current alternative to conventional agriculture embracing a systemic approach to food systems (as stated by proponents, whereas regulations do not include this approach strongly). Attempts to implement the agroecology worldview in practice, which is at the core of its systemic orientation, are recognised under the label of organic farming. Studies have shown the positive impacts in both countries ( Darnhofer, 2005; Schafer *et al.,* 2009). In Germany, the focus of policies is mainly on organic farming ( Lampkin *et al.,* 2020) as key informants pointed out for both countries. Neither agroecology nor organic agriculture can be summarised by a series of practices alone. Creating specific regulations for agroecology remains very questionable and is debated currently, as these would build on principles already adopted by organic agriculture and might open for greenwashing for larger food sector companies.

A possible resolution of the differences in interpretation of the concept of agroecology might be to raise the awareness of the knowledge–practice and the whole–parts dimensions. Agroecology understood as “the ecology of food systems” ( Francis *et al.,* 2003) and “a transdisciplinary, participatory and action-oriented approach” ( Méndez *et al.,* 2016) would then primarily be a field of knowledge, including normative principles and methodological approaches for describing, analysing and improving situations in practice. Agriculture, including certified organic agriculture, then obviously is practice in variable agreement with agroecological knowledge, principles and approaches. Agroecology, by definition, pertains to (agroeco)systems, which are wholes that express situation- and site-specific emergent properties because of their combination of interacting parts and, consequently, requires a “flickering” between focus on the whole and its parts to be understood and improved ( Bland and Bell, 2007). 

### Agroecology in science and practice

The importance of organic farming is also reflected in the number of published articles. There are more articles published (in English) in the last 30 years on organic agriculture than on agroecology. More papers using the term agroecology and food systems appeared in the last ten years, and there seems to be an emerging trend on the topic of agroforestry in Germany. Agroecology and organic agriculture regulations (EU regulations and IFOAM norms) have many common principles but diverge in some principles and practices ( Migliorini and Wezel, 2017). For the moment there are no agroecology regulations or norms at the European level. Instead of creating more regulations, INKOTA (2019) argue that the focus should be on the cessation of harmful policies.

Throughout the interviews, all key informants mentioned organic agriculture, either when referring to initiatives or when talking about implemented practices. Practices that have been listed were mostly practices defined as agroecological by Wezel *et al.* (2014). But flower strips and organic farming, most frequently mentioned by key informants, are not practices but, respectively, an agri-environmental measure which on its own is not sufficient and has to have a specific purpose linked to field and landscape management such as supporting the presence of natural enemies; and a concept and type of agriculture linked to a set of practices. In both countries, the integration of agroecological practices in the agricultural landscapes could not be quantified, and most key informants expressed the necessity to determine their integration. The potential of these practices in terms of broad implementation and promotion of sustainability should also be (re)assessed. As found in the previous mapping of Austria and for other countries such as Ireland, farmers have adopted different practices labelled as agroecological but do not name them this way ( Agroecology Europe, 2020). In order to render farming systems more resilient, a holistic approach is needed, meaning that practices need to be combined (implementing one cannot be considered as sufficient), assessed and adapted to local context.

Previous mapping in Austria ( Agroecology Europe, 2020) showed that regional differences (unfavourable conditions for intensification) and pioneers favoured the emergence of initiatives. In comparison to other European countries, farming in Austria is still small-scale but similarly to in other countries, farm numbers are decreasing while size is increasing.

### Diversity of agroecology initiatives

A new aspect of this study is the inclusion of living labs and the mapping of non-scientific training on agroecology in comparison to previous mapping projects ( Agroecology Europe, 2020; Balogh *et al.,* 2020). A key feature of living labs is “involving users as co-creators on equal ground” ( Almirall *et al.,* 2012). However, this was not the case of all self-proclaimed living labs but was found in other initiatives. As the concept of living labs, particularly in relation to agroecology, is not yet clearly defined ( McPhee *et al.,* 2021), the examples of the highlighted diverse initiatives represent an opportunity for stakeholders to assimilate the concept and discuss it in the light of agroecology.

Overall, the initiatives found in this work rarely called themselves an agroecology initiative, only four initiatives did. The others did not refute the term and when asked, identified with the agroecological worldview. This is likely due to their understanding of what agroecology is (often not seen as a movement or in the broader sense pertaining to whole food systems) and the specific focus on one aspect of the initiatives (e.g., food waste). However, all follow several agroecological principles, the most common ones being the co-creation of knowledge and the participation principle. Indeed, almost all initiatives interviewed create and share knowledge and aim to transform food systems. The previous mapping of Austria presented the main movement initiatives recognising and using the term (ÖBV-via Campesina Austria, Nyéléni Austria) ( Agroecology Europe, 2020).

The initiatives selected in this study, similarly to the ones in previous mapping efforts, encounter different barriers for their further development. Indeed, the question of continuity is key for lasting change. Projects are often limited in time and by the implication of people. Furthermore, financial means was often presented as a limiting factor by initiative representatives, as was also often mentioned in previous mapping report all over European initiatives. For example, the salary of the manager coordinating an “Öko-Modellregionen” in Bavaria is financed by the state of Bavaria for the first five years and then it goes through a regressive phase, which can lead to a different prioritisation of objectives (focus on local production and less on environmentally friendly production). Food policy councils could be considered as living labs with their purpose to democratise food systems, through horizontal governance, and aim to increase connectivity. At the moment, one of the limitations of the food policy councils studied here is the integration of farmers in these processes and recognition by authorities.

### Development of agroecology

Economic and political barriers were the most commonly identified hurdle for the development of agroecology, along with the missing recognition and awareness. These findings concur with many other studies ( Aare *et al.,* 2021; Ferrando *et al.,* 2021; Miles *et al.,* 2017). The failures of the current political framework have led scientists to propose ten action points to completely change the CAP ( Pe’er *et al.,* 2020). True cost accounting could be used to overcome the barrier of too low food prices, which are a consequence of externalisation of costs to the environment and society ( Benton and Bailey, 2019; HLPE, 2019). Even though the barriers are numerous and difficult to overcome, the recent crises (COVID-19 pandemic, the war in Ukraine) and climate change are playing a key role in awareness raising and changing of consumption habits. Last year, the European Committee of the regions adopted an opinion on agroecology ( Cros, 2021) as “the answer to Europe's agricultural, social and environmental challenges”. This still needs to be echoed in policies, especially in the national strategic plans of the new CAP.

### Methodological considerations

This study gives an overview of some of the existing initiatives and a partial view of the current state of agroecology in Austria and Germany as based on the key informants’ knowledge and readiness to respond. The information gathered from the key informants is based on their perceptions and interpretations, not necessarily on documented facts. This is a clear limitation of these kind of studies aiming to characterise the state of agroecology in terms of movement, policies, practices and research. A complementary approach for assessing the implementation of practice could have been to gather data from publications and look at the EU and state subventions for specific measures similar to the method used for estimating the silvopasture extent by Rodríguez-Rigueiro *et al.* (2021).

The interviews allowed to form a non-exhaustive yet illustrative list of initiatives showing that agroecology is gaining recognition, and that existing initiatives all work towards raising awareness going beyond Gliessman’s transition levels 1 and 2 ( Gliessman, 2007). Even though their impact is limited by their scale, their concrete goals promote the transformation and long-term success of the agroecological transition. In this study, the designation of agroecology initiatives was based on the information given by key informants. The initiative selection was not very strict as they only had to follow at least one agroecological principle to be considered. Agroecological principles give a framework within which the possible applications are very diverse, and it remains a challenge to properly fit the concept of agroecology to all kind of different initiatives. Furthermore, the purpose of this study was not to evaluate the initiatives. This could be done in a further step using other methodology such as the one developed by Dumont *et al.* (2021).

## Conclusion

Agroecology in Austria and Germany is recognised by few stakeholders, and the definition is subject to various interpretations. The use of the term is increasing, and different movements aim to spread the concept. In Austria and Germany, different policies promote organic farming and implicitly the implementation of agroecology. However, these are few and supported with insufficient funds. In both countries the research is too often fragmented, leading to very few advances in the development of agroecological practices. The effectiveness of specific practices and their interactions have to be tested in the three sustainability dimensions, i.e., their ecological, economic, and societal impacts.

The interviews showed that a clear understanding of the conceptual focus of agroecology, which in its essence is a holistic operationalisation of its principles in practice, is urgently needed. So far, research remains within disciplines, and a transdisciplinarity systems approach is broadly lacking when it comes to assessing the potential of agroecological approaches as the basis for the needed transformation of present agricultural and food systems.

In both countries, initiatives that work toward changing the food system according to agroecological principles have been developed. The emergence of initiatives is contingent to the geographic, economic, and political context. These initiatives have very specific focus, different ambition levels, and are at different levels of development. All can serve as examples for others and should be further evaluated in terms of their impacts and compliance with agroecological knowledge, principles, and methodological approaches. In both countries, movements are using the term agroecology often linked to food sovereignty, seed preservation, or specific practices like agroforestry. Living labs are also being developed to link different stakeholders and develop together processes to reach a common goal.

Comparing the state of agroecology-related initiatives in different countries and establishing clear criteria for assessing such initiatives will be crucial in the next years. In the last years, European countries have further developed the implementation of agroecology-related initiatives. The European ‘Partnership on agroecology living labs and research infrastructure’, if funded by the EU commission and European countries, will contribute to the urgently needed transition in the current agricultural system.

## Data Availability

Zenodo: Dataset - mapping of Agroecology in Austria and Germany. https://doi.org/10.5281/zenodo.7524270. ( Brumer *et al.,* 2023). The project contains the following underlying data: Brumer-et-al_Database_Austria-Germany.xlsm. (Anonymised responses for German and Austria interviews). Data are available under the terms of the Creative Commons Attribution 4.0 International license (CC-BY 4.0). Zenodo: AE4EU - Mapping questionnaire for key informant and initiative. https://doi.org/10.5281/zenodo.7520262. ( Grard *et al.,* 2023). This project contains the following extended data: Grard-et-al_AE4EU_Questionnaire_Key-informant_FV.pdf. (Blank English and German questionnaire used in this study). Data are available under the terms of the Creative Commons Attribution 4.0 International license (CC-BY 4.0).
