# Supplementary material for: Development of agroecology in Austria and Germany
Source: Open Res Eur. 2023 Feb 2;3:25. [Version 1] doi: 10.12688/openreseurope.15431.1 (PMC10445859; doi:10.12688/openreseurope.15431.1)
Supplement: Peer review history

# Peer review: Reviewer response for version 1

**DOI:** 10.21956/openreseurope.16682.r31144

**Published:** 2023-06-12

**Authors:** Andreas Thiel

**Affiliations:** 1 Section of International Agricultural Policy and Environmental Governance, Faculty of Organic Agricultural Sciences, Universitat Kassel, Kassel, Hesse, Germany

On the whole this is a useful although little surprising paper. My main critique would be an issue that is raised by the paper itself but that also undermines the paper: The lack of a definitional effort concerning agroecology (AE). For the purposes of the paper at least certain core dimensions of AE should be indicated in order to sort what the paper considers part of AE and what it does not consider part of AE. The vagueness of what AE is leads to a lot of imprecisions in the paper. Only to name one, the paper and respondents consider organic agriculture (OA) as a version of AE.

 However, the paper also reports that interviewees critique a lack of policies and support for AE. As a matter of fact, policies on OA and financial support are very much existent. Thus, the paper is misleading in this regard. In my view a definition of AE should define dimensions and “types of AE”. In the context of such a definition OA would maybe not be equivalent of AE but it would fulfill some of its dimensions. Correspondingly, interviewees and authors would be able to report that policies on OA exist, for example, but not on AE proper, which is the case.

 The reflection of how understandings of interviewees relate to their professional context could be advanced earlier in the paper as this obvious point otherwise may fall under the table.

 In relation to the table on relevant courses and initiatives, it is not clear to me how these were identified. A lot seems to be missing and distinguishing criteria are not clear. Maybe a clearer definition of AE could also help in that respect?

 The transformative dimension of AE deserves more attention and emphasis in the paper, in my view.

 The treatment of living labs and initiatives should be elaborated also in terms of what distinguishes these as well as how makes for an AE initiative and what does not.

 The quantitative analysis of wordings misses an interpretation, it may better be left out in my view.

 If data was sufficient, it would be useful to better understand the current politics around the framing of AE in relation to OA, regenerative agriculture and the like.

Is the study design appropriate and does the work have academic merit?

Partly

Is the work clearly and accurately presented and does it cite the current literature?

Yes

If applicable, is the statistical analysis and its interpretation appropriate?

Partly

Are all the source data underlying the results available to ensure full reproducibility?

Partly

Are the conclusions drawn adequately supported by the results?

Partly

Are sufficient details of methods and analysis provided to allow replication by others?

Yes

Reviewer Expertise:

Agricultural policy

I confirm that I have read this submission and believe that I have an appropriate level of expertise to confirm that it is of an acceptable scientific standard, however I have significant reservations, as outlined above.

# Peer review: Reviewer response for version 1

**DOI:** 10.21956/openreseurope.16682.r31143

**Published:** 2023-05-16

**Authors:** Nicola Randall

**Affiliations:** 1 Department of Agriculture and Environment, Harper Adams University, Newport, England, UK

There is a comprehensive background, that introduces the concept of agroecology and differing approaches, together with some existing sources. The background introduces the purpose of the project, with clear objectives. 

**Methods:**

*Literature review:*

 It is good to see the keywords that were used in the methods. It would be useful to state on what dates these searches were carried out, & also whether any test searches were carried out (eg to see if adding Germany & Austria to the topic searches restricted the results or not). The last line before the subheading 'interviews' states that an effort was made to find initiatives in different regions, but it is not clear how this was carried out.

*Interviews:*

 It is useful to see the underlying questionnaires.

**Results & conclusions:**

 The results are primarily based on the interviews with a brief overview of the literature review findings. From what I can see, the summaries of key points from the interviews are clear, with some specific examples, and these lead to logical conclusions.

Is the study design appropriate and does the work have academic merit?

Yes

Is the work clearly and accurately presented and does it cite the current literature?

Yes

If applicable, is the statistical analysis and its interpretation appropriate?

Not applicable

Are all the source data underlying the results available to ensure full reproducibility?

Yes

Are the conclusions drawn adequately supported by the results?

Yes

Are sufficient details of methods and analysis provided to allow replication by others?

Yes

Reviewer Expertise:

Agroecology. Evidence synthesis

I confirm that I have read this submission and believe that I have an appropriate level of expertise to confirm that it is of an acceptable scientific standard.

# Peer review: Reviewer response for version 1

**DOI:** 10.21956/openreseurope.16682.r30798

**Published:** 2023-02-20

**Authors:** Felipe Gallardo López

**Affiliations:** 1 Graduate College Campus Veracruz (Colegio de Postgraduados Campus Veracruz), Veracruz, Mexico

**In general:**

 The document makes a good contribution to current scientific, practical and discursive state in relation to different manifestations of agroecology in Austria and Germany. It identifies its limitations to achieve the agroecological transition. The results are useful for the design and application of the policy in these two countries. The document raises the need to evaluate initiatives related to agroecology with clear criteria. In this sense, I would propose to evaluate them from the perspective of agroecological principles in their design, in their practice and in the speeches of their actors to find dissonances or consonances, as well as distances in compliance with the principles. It is suggested to better define the general and particular objective and describe it only once in the introduction and in the summary. It is suggested to improve the description of the methodology.

**Specific observations:**

 The description in the text of figure 2 indicates that there were 5 steps. However, only four are shown in the figure. I think that the fifth is missing and I recommend that it be in contrast to the two countries analysed.

 The document shows the objectives in four moments. One in the summary, and three in the introduction, considering that the answers to the questions raised are objective. Although, in the approaches of these four moments they are not exclusive, it is not clear what the central and particular objective is. I suggest building a single general one and perhaps some particular ones if necessary, but that it be raised only once in the introduction and once in the summary. A particular objective addressed was to identify the constraints for the agroecological transition and they are not included in the objectives, I suggest including it.

 Describe the methodological approach synthetically (in the summary) and broadly in the methodology section in the same order as in Figure 2.

 The first paragraph of results is practically methodology.

 Remove the last paragraph of conclusions which refers to the financing of living laboratories.

Is the study design appropriate and does the work have academic merit?

Yes

Is the work clearly and accurately presented and does it cite the current literature?

Yes

If applicable, is the statistical analysis and its interpretation appropriate?

Yes

Are all the source data underlying the results available to ensure full reproducibility?

Yes

Are the conclusions drawn adequately supported by the results?

Partly

Are sufficient details of methods and analysis provided to allow replication by others?

Yes

Reviewer Expertise:

Agroecosystem

I confirm that I have read this submission and believe that I have an appropriate level of expertise to confirm that it is of an acceptable scientific standard.
